# Construction of an index system for evaluating the effectiveness of transitional care in kidney transplant recipients

**DOI:** 10.1186/s12911-021-01496-9

**Published:** 2021-04-19

**Authors:** Xinyi Zhou, Ping Ding, Qiaolan Yang, Ping Wang, Haimei Zhou, Jing Fu, Dongrui Miao

**Affiliations:** 1 The First Affiliated Hospital of Anhui Medical University, Hefei, Anhui Province People’s Republic of China; 2 Department of Nosocomial Infection Management, The First Affiliated Hospital of Anhui Medical University, Hefei, Anhui Province People’s Republic of China; 3 Department of Urology, The First Affiliated Hospital of Anhui Medical University, Hefei, Anhui Province People’s Republic of China; 4 Department of Infectious Diseases, The First Affiliated Hospital of Anhui Medical University, Hefei, Anhui Province People’s Republic of China

**Keywords:** Transitional care, An evaluation index system, Delphi survey technique, Omaha system, Kidney transplant recipients

## Abstract

**Background:**

Previous studies showed that transitional care reduces the complication rate and readmission rate and improves the quality of life in kidney transplant receipts, nevertheless, in fact there are no standard evaluation indexes and debatable scientific of existing indexes in kidney transplant recipients. Therefore, the aim of this study was to construct an evaluation index system to assess the effects of transitional care in kidney transplant recipients.

**Methods:**

Based on Omaha system, an initial evaluation index system about the effects of transitional care in kidney transplant recipients was drafted by the literature review and semi-structured interview. Two rounds of correspondence were conducted in 19 experts and the analytic hierarchy process (AHP) was used to calculate the weights of all indexes.

**Results:**

Five first-level indexes, sixteen second-level indexes, and forty-eight third-level indexes were selected in the initial evaluation index system. The authority coefficient of two-round expert consultations was 0.90 and coordination coefficients of indexes ranged from 0.24 to 0.34.

**Conclusion:**

The established evaluation index system for the effectiveness of transitional care for kidney transplant recipients was scientific and reliable. Furthermore, it would be a potential method to evaluate effects of transitional care in kidney transplant recipients after further examination.

**Supplementary Information:**

The online version contains supplementary material available at 10.1186/s12911-021-01496-9.

## Background

Kidney transplantation is currently the optimal kidney replacement therapy for the treatment of patients with advanced kidney disease compared to haemodialysis and peritoneal dialysis [1]. In 2018, the annual number of kidney transplant recipients in China was 13,029 [2]. After kidney transplantation, patients should take medication for their whole life, nevertheless, complex medication regimens [3], medication-related side effects [4], and the lack of professional self-care knowledge and guidance in these patients [5], could lead multi-complications [6] and increase readmission rates [5], furthermore, impair the Quality of Life (QoL) [7].

Previous studies showed that transitional care could reduce the complication rate and readmission rate and improve the quality of life in kidney transplant [8, 9]. High-quality indexes for effectiveness evaluation could promote quality of nursing care because it was not only to assess the quality of transitional care but also to identify the insufficiencies in existing transitional care. However, at present, there are no standard evaluation indexes and debatable scientific of existing indexes in chronic disease transitional care in China [10]. Some researchers have explored the construction of a standardized evaluation index system in people with stroke [11], coronary heart disease [12] and other diseases, and some researchers have also explored the construction of a transitional care evaluation index system for the entire chronic disease population [13]. However, in the field of kidney transplant recipients, a standardized and systematic index system for evaluating the effectiveness of transitional care has not been developed, and the index system for evaluating the chronic disease population is not disease-specific and lacks evidence-based studies.

Therefore, present study used the Omaha system as the theoretical framework and Delphi method combined with analytic hierarchy process (AHP) to construct an index system for evaluating the effectiveness of transitional care in kidney transplant recipients.

## Methods

### Objective

The objective of this study is to provide a reference for scientific and effective evaluation of transitional care in kidney transplant receipeints and promote the improvement of nursing quality.

### Description of a research group

There were six team members in the research group, including two graduate nursing students, one associate professor of kidney transplant nursing research, one director of the kidney transplant unit, one head nurse of the kidney transplant unit, and one expert related to scale statistics. The main tasks of the research team included: review of relevant literature, preliminary construction of the evaluation index entry pool, selection of experts, preparation and distribution of expert correspondence forms, and collation and analysis of expert opinions.

### Inclusion criteria of correspondence experts

Experts were selected by snowball sampling method in China. Inclusion criteria for experts were: (1) Nursing experts: Intermediate or above title, who have conducted research related to kidney transplantation and/or transitional care; 10 years or more in the field of nursing clinical/management/psychology/education; bachelor's degree or above; (2) Clinical medicine experts: above intermediate title, 10 years or more in kidney transplantation clinical work; master's degree or above; (3) All included experts were interested in supporting the study and could guarantee sustained attendance for the duration of the present study.

### Development of evaluation indicators system

An initial effectiveness evaluation index system consisting of 5 primary indicators, 19 secondary indicators, and 61 tertiary indicators was developed through literature review and patient interviews.

#### Systematic literature reviews

The keywords "renal transplant*/kidney transplant*/renal transplant recipients/kidney transplant recipients, transitional care/continuity of care/continuing nursing/patient discharge planning/discharge planning/telemedicine/comprehensive care/multidisciplinary care" were searched in electronics database. Based on the systematic literature review, the Omaha system was used as the theoretical framework, and 42 questions in four major domains were referred to the Omaha problem classification system, which were combined with the effectiveness evaluation indexes and disease characteristics in the literature to form a preliminary pool of entries for evaluating the effectiveness of transitional care for kidney transplant recipients.

#### Patient interviews

A comprehensive understanding of kidney transplant recipients' experiences of transitional care and the indicators of transitional care effectiveness that transplant recipients consider important were obtained through interviews with transplant recipients, and the views of transplant recipients were considered in the construction of the indexes of transitional care effectiveness. Patients for this interview had to be 18 years or older. All patients provided their written informed consent in accordance with the Helsinki declaration.

Eleven selected patients were interviewed after their clinic follow-up. The outline of the interview included: (1) What is your current health status (physical symptoms, mental status and social activities)? (2) What are the indicators that concern you when you get the test report at the follow-up? (3) What changes have occurred in your habits, social activities, and perception of the disease after the transplant? (4) What are the issues that were important to you since your discharge from the hospital but were ignored by the medical staff?

#### Correspondence from experts

Expert correspondence form included 3 parts. First, the purpose, significance, theoretical basis and related concepts of present study; second, content consultation: consisting of indicators and scores, experts  were invited to rate the importance of the entries, based on the Likert 5-point scale, from "unimportant" to "very important". Each level of indicators was left a blank column for additional description and recommendations; third, expert authority scale: including basic information of experts, questionnaire of judgment basis and questionnaire of content familiarity. The questionnaires were distributed by both email and on-site research. Experts of correspondence had to be 18 years or older.

In present study, indicators with a mean importance assignment > 4 and a coefficient of variation < 0.2 [14] were retained, and the indicators were selected by combining both expert opinions and discussions of the project team.

### Statistical methods

SPSS 16.0 software (SPSS Inc., Chicago, IL, USA) was used for data analysis. The general profile of the experts was expressed by frequency. The degree of expert opinion coordination was expressed as coefficient of variation (CV) and coordination coefficient (Kendall's W). The expert positivity coefficient was expressed by the questionnaire recall rate. The degree of expert authority was expressed by the expert authority coefficient (Cr), Cr = (familiarity coefficient + judgment basis coefficient)/2 [15]. Familiarity was categorized as unfamiliar (0.2 points), somewhat unfamiliar (0.4 points), somewhat familiar (0.6 points), very familiar (0.8 points), and proficient (1.0 points) [16]. Judgment score was assessed according a previous study [17]. Yaaph10.0 software (AHP program) was used to calculate the weight and consistency ratio (CR) of each index. The combination weight is calculated by applying the multiplicative method, that is, the product of the original weight of the indicator within the level and the combination weight of its higher-level indicators. For a level 1 indicator, the original weight is the combination weight.The weight of the indicator represents the relative importance of the indicator in the overall indicator system [18, 19]. Generally, the more important the evaluation indicator is, the higher the weight value is.

## Results

### Correspondence experts involved

#### Basic information

Twenty-one experts were initially selected as correspondence experts, and after communication finally 19 experts participated in the study. These experts were from 10 tertiary hospitals and 1 higher education institution in 8 provinces. Demographic data on gender, age, work experience, academic degree, job title, and posts of participated experts had been summarized in Table 1.

**Table 1:** Profile of expert’s information

Items	N (%)
Gender	
Male	5 (26.3)
Female	14 (73.7)
Age (Year)	
≤ 40	5 (26.3)
41–50	8 (42.1)
≥ 51	6 (31.6)
Work experience	
10–20	13 (68.4)
21–30	5 (26.3)
≥ 31	1 (5.3)
Academic degree	
Bachelor's degree	11 (57.9)
Master's degree	3 (15.8)
Ph.D degree	5 (26.3)
Job title	
Intermediate	6 (31.6)
Associate senior	7 (36.8)
Senior	6 (31.6)
Posts	
Professor/dean, school of nursing	2 (10.5)
Director of nursing	10 (52.6)
Division director	5 (26.3)
Clinical nurse	2 (10.5)

#### Positive level of experts

In the first-round, twenty-one (n = 21) questionnaires were distributed and 19 were returned, with a recall rate of 90.48%. In the second-round, nineteen (n = 19) questionnaires were distributed and 19 were returned, with a recall rate of 100%.

#### Degree of authority of experts

The coefficient of expert familiarity (Cs) was 0.89 ± 0.14, the coefficient of judgment basis (Ca) was 0.90 ± 0.07, and the coefficient of authority (Cr) was 0.90 ± 0.09.

#### The degree of expert coordination

The Kendall's consistency coefficients of the first-round experts for the primary, secondary, tertiary and overall indicators were 0.20 (*p* = 0.004), 0.24 (*p* < 0.001), 0.27 (*p* < 0.001) and 0.25 (*p* < 0.001), respectively (Table 2). The Kendall's consistency coefficients of the second round of experts for the primary, secondary, tertiary and overall indicators were 0.24 (*p* = 0.001), 0.34 (*p* < 0.001), 0.24 (*p* < 0.001) and 0.24 (*p* < 0.001), respectively (Table 2).

**Table 2:** Summary of Kendall’s Concordance Coefficient W

	Primary indicators	Secondary indicators	Tertiary indicators	Total
First-round				
Kendall’s W	0.20	0.24	0.27	0.25
χ^2^	15.14	83.57	307.64	404.43
* p*	0.004	< 0.001	< 0.001	< 0.001
Second-round 2				
Kendall’s W	0.24	0.34	0.24	0.24
χ^2^	17.85	95.84	229.45	327.47
* p*	0.001	< 0.001	< 0.001	< 0.001

### Results of expert consultation

In the first-round of expert consultation, the mean of the primary indicators was 4.26 to 4.84, and the coefficient of variation was 0.10 to 0.17; the mean of the secondary indicators was 3.89 to 4.95, and the coefficient of variation was 0.05 to 0.24; the mean of the tertiary indicators was 3.63 to 4.95, and the coefficient of variation was 0.05 to 0.23.

According to the selection criteria and experts' opinions, after the discussion of the research group, 2 indexes were revised in the first-level indexes. 2 indexes were added, 5 indexes were deleted, and 2 indexes were revised in the second-level indexes. 14 indexes were deleted, 5 indexes were added and 2 indexes were revised in the third-level indexes (Table 3).

**Table 3:** Details of modifications of indexes after first-round of consultation

	Original indexes	Decision by the research group	Revised indexes
1st level indexes	Environmental domains	Revised	Social and Environmental Domains
	Psychosocial domains		Psychological Domains
2nd level indexes	Not available	Added	Positive mental state
			Negative mental state
	Surrounding/work environment	Deleted	Not available
	Mental health		
	Cognitive function		
	Neuromusculoskeletal function		
	Sexual function		
	Medication	Revised	Medication adherence
	Regular monitoring		Regular follow-up and self-monitoring
3rd level indexes	Not available	Added	Number of unscheduled readmissions after discharge from hospital
			I can tolerate and maintain a good attitude in the face of possible post-transplant problems
			Do you have a positive attitude about your current physical condition
			Be able to consult a professional before vaccination
			Follow up with your doctor and have a physical examination as required (every six months to a year, as required by your transplant surgeon)
	There are air disinfection machines that match the function of the living area and can disinfect the dwelling as required	Deleted	Not available
	Presence of high pollution levels in water or air (smelting, power and other industries nearby)		
	The presence of infectious substances in the work environment (such as dust, yeast, etc.)		
	Worries		
	Susceptible to fatigue		
	Urine nitrogen		
	Estimated glomerular filtration rate		
	Memory loss		
	Joint pain		
	Trembling hands		
	Difficulty concentrating		
	Sexual dysfunction		
	Women of child-bearing age know the issues related to fertility after transplantation		
	Knowing the importance of following medical advice on medication use		
	Insomnia	Revised	Sleep status
	Knowledge of precautionary measures for various infections		To know the precautions for lung infection and urinary tract infection

After the second-round of consultation, the mean of primary indicators varied from 4.05 to 4.74 with coefficients of variation of 0.10 to 0.15; the mean of secondary indicators varied from 3.63 to 4.95 with coefficients of variation of 0.05 to 0.25; the mean of tertiary indicators varied from 3.95 to 5.00 with coefficients of variation of 0.00 to 0.21. After the research group’s discussion, 2 indictors in the second-level indexes were added and 4 tertiary indicators were deleted (Table 4). Details of the result of two rounds of correspondence refer to Additional file 1.

**Table 4:** Details of modifications of indexes after second-round of consultation

	Original indexes	Decision by the research group	Revised indexes
2nd level indexes	Social activities	Revised	Social function
	Relationship with medical resources		Healthcare resource utilization
3rd level indexes	Participation in work (when condition permits)	Deleted	Not available
	Uric acid		
	Knowing the importance of regular monitoring of tumor markers		
	Overall effect of transitional care		

The final evaluation index system of 5 first-level indexes, 16 second-level indexes, and 48 third-level indexes for the evaluation of the effectiveness of kidney transplant recipients transitional care is shown in Table 5. The consistency test CR values for the total ranking of first-, second-, and third-level indexes ranged from 0.000 to 0.05, all < 0.1 [20].

**Table 5:** Evaluation of the effectiveness of traditional care in kidney transplant recipients

Indicators	Score	CV	Weights
1. Physiological Domains	4.74 ± 0.45	0.10	0.30
1.1. Transplanted kidney function	4.95 ± 0.23	0.05	0.13
1.2. Infection status	4.84 ± 0.38	0.08	0.10
1.3. Cardiovascular function	4.53 ± 0.61	0.14	0.05
1.4. Digestive function	4.26 ± 0.81	0.19	0.02
1.1.1. Creatinine	4.95 ± 0.23	0.05	0.09
1.1.2. Be aware of the signs of rejection and inform the health care provider in a timely manner	4.84 ± 0.50	0.10	0.05
1.2.1. History of post-discharge infections requiring in-patient treatment (lung infections, urinary tract infections, etc.)	4.74 ± 0.56	0.12	0.02
1.2.2. To know the symptoms of infection and seek medical attention soon	4.84 ± 0.38	0.08	0.05
1.2.3. To know the precautions for lung infection and urinary tract infection	4.79 ± 0.42	0.09	0.03
1.3.1. Blood pressure	4.58 ± 0.61	0.13	0.03
1.3.2. Be aware of the importance of managing blood pressure and lipids and the normal values of the indicators	4.53 ± 0.51	0.11	0.02
1.3.3. Overall cholesterol level	4.11 ± 0.66	0.16	0.01
1.4.1. Diarrhea occurred after discharge from the hospital	4.53 ± 0.61	0.14	0.01
1.4.2. To know the simple management of diarrhea and nausea/vomiting	4.32 ± 0.67	0.16	0.01
1.4.3. Nausea/vomiting after discharging from hospital	3.95 ± 0.71	0.18	0.003
2. Psychological Domains	4.74 ± 0.56	0.12	0.30
2.1. Positive mental state	4.84 ± 0.38	0.08	0.22
2.2. Negative mental state	4.42 ± 0.61	0.14	0.08
2.1.1. I am fully confident about my future life	4.74 ± 0.45	0.10	0.11
2.1.2. I can tolerate and maintain a good attitude in the face of possible post-transplant problems (elevated creatinine, lung infections, etc.)	4.63 ± 0.50	0.11	0.06
2.1.3. Do you have a positive attitude about your current physical condition	4.63 ± 0.50	0.11	0.06
2.2.1. Anxiety/depression (due to appearance, body shape changes, complications, secondary transplants, costs, etc.)	4.47 ± 0.61	0.14	0.05
2.2.2. Be well-informed about solutions for anxiety alleviation	4.32 ± 0.67	0.16	0.03
3. Health Behaviour-related Domains	4.63 ± 0.45	0.11	0.20
3.1. Medication adherence	4.95 ± 0.23	0.05	0.07
3.2. Regular follow-up and self-monitoring	4.95 ± 0.23	0.05	0.07
3.3. Nutritional status	4.58 ± 0.61	0.13	0.03
3.4. Physical activity status	4.37 ± 0.60	0.14	0.02
3.5. Lifestyle habits	4.53 ± 0.51	0.11	0.02
3.1.1. Be able to follow medical advice and use medication accurately (ensure the frequency, dosage and duration of medication and do not stop medication without permission)	5.00 ± 0.00	0.00	0.03
3.1.2. Do not take medication without the consent of a professional	4.79 ± 0.42	0.09	0.02
3.1.3. Be aware of the possible side effects of taking immunosuppressive drugs	4.68 ± 0.48	0.10	0.01
3.1.4. Be able to consult a professional before vaccination	4.63 ± 0.60	0.13	0.01
3.2.1. To learn proper self-monitoring (e.g. blood pressure, temperature, weight, urine output)	4.79 ± 0.42	0.09	0.03
3.2.2. Actively monitor indicators (e.g. blood pressure, temperature, weight, urine output) and know normal values	4.74 ± 0.45	0.10	0.02
3.2.3. Follow up with your doctor and have a physical examination as required (every six months to a year, as required by your transplant surgeon)	4.74 ± 0.45	0.10	0.02
3.2.4. Number of unscheduled readmissions after discharge from hospital	4.16 ± 0.77	0.18	0.01
3.3.1. Blood glucose	4.53 ± 0.51	0.11	0.01
3.3.2. Be able to opt for healthy foods and maintain a balanced diet (small, frequent meals, high quality protein, high calcium, low cholesterol, etc.)	4.53 ± 0.70	0.15	0.01
3.3.3. Be able to avoid forbidden foods such as grapefruit and immune-enhancing health products such as ginseng, deer antler, royal jelly, etc	4.90 ± 0.32	0.06	0.01
3.3.4. Body mass index	4.26 ± 0.65	0.15	0.002
3.4.1. Self-care status	4.32 ± 0.58	0.13	0.01
3.4.2. To know the type and amount of exercise that is appropriate for you at different stages after surgery	4.47 ± 0.51	0.11	0.01
3.4.3. Appropriate exercise program in place and executed	4.11 ± 0.66	0.16	0.003
3.4.4. To know the precautions for sex after transplantation	4.05 ± 0.85	0.21	0.002
3.5.1. Quit smoking and restricted drinking	4.63 ± 0.68	0.15	0.01
3.5.2. Regularity of routine	4.63 ± 0.60	0.13	0.01
3.5.3. Sleep status	4.63 ± 0.50	0.11	0.01
4. Social and Environment Domains	4.53 ± 0.51	0.11	0.14
4.1. Healthcare resource utilization	4.37 ± 0.76	0.17	0.08
4.2. Life environment	4.26 ± 0.65	0.15	0.05
4.3. Social function	3.63 ± 0.90	0.25	0.02
4.1.1. Be able to access solutions to post-transplant related questions and assistance from the hospital/community/public media (e.g. WeChat, etc.)	4.68 ± 0.58	0.12	0.04
4.1.2. Be supported and assisted by disease-related national medical policies (e.g., health insurance, chronic disease and serious illness assistance, etc.)	4.47 ± 0.61	0.14	0.02
4.1.3. Maintain favorable communication with health care professionals	4.58 ± 0.61	0.13	0.02
4.2.1. The residential environment is clean, tidy and well ventilated	4.68 ± 0.48	0.1	0.04
4.2.2. No allergenic substances (such as pollen, willow, animal hair, etc.) in the residential and occupational environment	4.16 ± 0.69	0.17	0.01
4.3.1. Be able to obtain the support of family and friends	4.74 ± 0.45	0.1	0.01
4.3.2. Be able to engage in normal social and leisure activities (if condition permits) (doing household chores, visiting friends and relatives, dining out, shopping, vacation, etc.)	4.11 ± 0.57	0.14	0.003
4.3.3. Sharing experiences, questions and knowledge about kidney transplantation with fellow patients	4.05 ± 0.71	0.17	0.002
5. Satisfaction	4.05 ± 0.62	0.15	0.06
5.1. Transitional care services	4.68 ± 0.48	0.10	0.05
5.2. Transitional care experience	4.21 ± 0.63	0.15	0.02
5.1.1. Transitional care formats (e.g., mini-lectures, renal club, WeChat groups, brochures, micro-lessons, etc.)	4.68 ± 0.48	0.10	0.02
5.1.2. Transitional care content (e.g., diet, exercise, drug knowledge, psychological support, etc.)	4.58 ± 0.51	0.11	0.01
5.1.3. Consistent information from health care workers	4.42 ± 0.69	0.16	0.01
5.2.1. Promptness, persistence and availability of transitional care	4.79 ± 0.42	0.09	0.01
5.2.1. Attitudes of service when providing care	4.47 ± 0.61	0.14	0.004

Data was presented as mean ± standard deviation (SD), CV = Coefficient of variation

## Discussion

### Analysis of the scientific and reliability of the evaluation index system

The Omaha system is one of the standardized languages of nursing recognized by the American Nurses Association and includes three parts, which are an assessment component (Problem Classification Scheme), a care plan/services component (Intervention Scheme), and an evaluation component (Problem Rating Scale for Outcomes). Among them, the problem classification scheme includes a total of 42 common problems in four domains: environmental, psychosocial, physical and health-related behaviors. Wong et al. (2004) found that a health status record form of erminally ill patients established by a modified Omaha's problem classification scheme could assess the effectiveness of care from various aspects of environmental psychological and physiological behaviors [21]. Wei et al. (2019) formulated a transitional care evaluation system for breast cancer patients based on literature research, combined with Omaha's system, expert meetings, and through Delphi consultation [22]. The preliminary application showed the practicality and scientific validity of the system. Therefore, the Omaha system can be used for the construction of the evaluation index system and can reflect the problem more comprehensively, and the choice of the theoretical framework of this study is scientific. The scientific and disease-specific nature of the index system was fully reflected through an extensive literature review of literature related to transitional care interventions for kidney transplant recipients, extraction of effectiveness evaluation indicators, semi-structured interviews with transplant recipients, clarification of transplant recipients' experiences and expectations of transitional care, and supplementation of the index system content.

In this study, based on modified Delphi method, selected correspondence experts were from 8 provinces across China, including nurse leaders/nurses of kidney transplantation units, department vice directors/directors, and professors of colleges, who had unique insights into kidney transplantation transitional care and follow-up work, and had more in-depth studies on the Omaha system, with good representation of experts; the recall rates of the two rounds of correspondence questionnaires were 90.5% and 100%, indicating that the experts were more active in this study. The Cr value of the expert consultation was 0.90, reflecting the higher authority of the experts; the experts in the two rounds of consultation had significant agreement on the included indicators (all *p* < 0.05), and the consultation results were credible. In order to make the subjective data of expert judgment more scientific, hierarchical analysis was used to statistically process the expert judgment. Hierarchical analysis is a multi-criteria decision-making thinking that combines qualitative and quantitative wants, which could be compatible with the Delphi survey technique to solute problems in term of multi-level indicator systems or decision that can not be soluted by quantitatively [23]. In this study, the consistency test was used to assess the logical consistency of the judgment matrix and was expressed as the CR. It was generally considered that if CR < 0.1, the degree of inconsistency of the judgment matrix was within the accepted range, indicating that the weights of indicators at all levels were acceptable.

### Content analysis of the evaluation index system

The final evaluation index system of the effectiveness of transitional care for kidney transplant recipients in this study included 5 first-level indexes, 16 second-level indexes, and 48 third-level indexes. The primary indicators are ranked by weight: physiological domains = psychological domains > health behavior-related domains > social environment domains > satisfaction.

The highest weight coefficients of 0.30 were assigned to the physiological and psychological domains, indicating that experts considered the physiological and psychological domains to be equally important in evaluating the effectiveness of transitional care in kidney transplantation. Wang et al. (2017) found that the health outcomes of patients with chronic diseases were the most important evaluation index of the effectiveness of transitional care. The indexes of both physical and psychological domains constructed in present study were health outcomes, which are consistent with their findings [13].

In the physiological domains, the weight of second-level indexes "transplant kidney function" (weight = 0.13) and "infection status" (weight = 0.10) were the highest two indexes, indicating that these two indexs are critical to the physical health outcomes of transplant recipients. Transplant kidney function directly associated with the condition of the transplanted kidney and predict the survival of the transplanted kidney [24], therefore, maintaining transplanted kidney function was the primary objective of transitional care and transplant kidney function was the indicator of evaluating the effectiveness of transitional care. Transplant recipients were highly susceptible to infections due to the effects of immunosuppression. Studies had shown that approximately 80% of recipients had at least one infection within 1 year after kidney transplantation, with pulmonary and urinary tract infections being the main types of infections [25, 26]. It is suggested that the education of transplant recipients on the recognition of early symptoms of infection and infection precautions in the transitional care should be strengthen.

The highest weight value (weight = 0.22) was assigned to the secondary indicator "positive psychological state" in the psychological domains, indicating that the experts considered the positive psychological state of the transplant recipient to be important. The postoperative psychological state of kidney transplant recipients was greatly influenced by the functional state of the transplanted organ in vivo and was prone to more psychological problems [27]. Along with the development of positive psychology, the guidance of positive emotions in transplant recipients should be strengthened in transitional care, and the development of positive psychological qualities in kidney transplant recipients should be emphasized to promote physical and mental health [28].

The weighting coefficient of the health behavior-related domains was 0.20, which play an important role in the evaluation of the effectiveness of transitional care. In present study, the health-related behavior domains of the Omaha problem classification system were adjusted and integrated with disease characteristics, and the results showed that the secondary indicators "medication-related adherence" (weight = 0.07) and "regular follow-up and self-monitoring" (weight = 0.07) were equally important in the health-related behavior domains. The regular long-term administration of immunosuppressive drugs and regular outpatient follow-up in kidney transplant recipients are the two main features that distinguish them from other surgical patients [29]. Kidney transplant recipients need to take immunosuppressive drugs for life, and  the study has shown [30] that the longer the post-transplant period, the poorer the adherence to immunosuppressive drugs in kidney transplant recipients, and that poor adherence can lead to rejection and failure of the transplanted kidney. Therefore, improving medication adherence in transplant recipients is an important goal of transitional care and medication adherence is an important indicator of outcome evaluation. Long-term use of immunosuppressive drugs is a risk factor for new complications such as diabetes and pulmonary infections after transplantation [26, 31], and regular follow-up and self-monitoring of transplant recipients should be emphasized to achieve early detection and diagnosis as well as early treatment. It was reminded that we should strengthen the education and training of transplant recipients' self-monitoring ability to improve their self-management awareness and ability in the transitional care.

The present study was combined the assessment of social health and the assessment of the environment, which was different from Omaha system. After discussion with experts and reviewing the literature, it was concluded that the first-level index of social environment could include the living environment, health care resource utilization, and social function [32, 33]. In this study, the former secondary indicator "social activities" should have been removed according to the screening criteria. However, drug side effects and complications can impair a kidney transplant recipient's ability to participate in daily social and recreational activities, so social activity is a crucial outcome for kidney transplant recipients because it is a predictor of kidney transplant recipients' ability to return to daily life [34]. Two clinical care specialists were called for further consultation and agreed to retain and recommend modification to social function. This indicator was retained and renamed to "social function" in accordance with the experts' opinion.

The weighting coefficient of satisfaction was 0.06, indicating that experts considered satisfaction to be less important than other level 1 indicators, which may be related to the subjective nature of satisfaction. One expert stated that patient experience should be distinguished from quality of care and that the level of care should not be too dependent on the subjective feelings of patients.

In addition, present study was constructed as a gross indicator, and the appropriate indicators or weights should be adjusted for transplant recipients of different ages. For example, elderly transplant recipients have a higher incidence of post-transplant diabetes mellitus [35], lower medication adherence [36], and greater susceptibility to infection [37] compared to young and middle-aged transplant recipients, so the weight of indicator "blood glucose" may need to be higher for elderly transplant recipients, and the weight of indicators related to medical resource utilization, family and social support, and medication adherence may be higher. Also, indicators related to the assessment of frailty and cognitive impairment may be increased. Based on the present study, the weighting of the indicators may be increased or decreased for different age groups in order to better match the clinical application.

### Limitations

Expert consultation has the limitation of population selection, and this study has tried to select representative experts as much as possible, but it is undeniable that a different group of experts may get different results. This study can only represent the importance of the selected experts for the indicators considered.

## Conclusion

This study used the Omaha system as the theoretical framework, combined with literature review, semi-structured interviews, expert consultation and hierarchical analysis to construct a scientific and systematic index system for evaluating the effectiveness of transitional care for kidney transplant recipients. This index system also provides the evidence for nursing staff to determine the priority of transitional care when making decision. In future, a preliminary clinical experiment is going to be conducted to examine the effects of this index system on transitional care in kidney transplant recipients.

## Supplementary Information

**Additional file 1**. Summary of the result of two rounds of correspondence.

## Data Availability

The dataset supporting the conclusions of this article is not publicly available due to the privacy of participants is included but are available from the corresponding author on reasonable request.

## Abbreviations

AHP: Analytic hierarchy process
Ca: The coefficient of judgment basis
Cr: Authority coefficient
CR: Consistency ratio
Cs: Coefficient of expert familiarity
CV: Coefficient of variation
QoL: Quality of life
SD: Standard deviation
